# Optimal Horseshoe Crab Blood Collection Solution That Inhibits Cellular Exocytosis and Improves Production Yield of Limulus Amoebocyte Lysate for Use in Endotoxin Tests

**DOI:** 10.3390/ijms26146642

**Published:** 2025-07-11

**Authors:** Mengmeng Zhang, Sophia Zhang, Jessica Zhang

**Affiliations:** Department of Biology, Westford Academy, Westford, MA 01886, USA; szhang6781@gmail.com (S.Z.); jzhang89123@gmail.com (J.Z.)

**Keywords:** limulus amoebocyte lysate (LAL) assays, endotoxin, PBS–caffeine, exocytosis, chromogenic assay

## Abstract

Limulus amoebocyte lysate (LAL) assays have emerged as among the most effective approaches for detecting endotoxins and fungi in vitro since they were first tested 50 years ago. Although detailed protocols are publicly available, conventional LAL collection methods (3% sodium chloride) waste as much as 80% of the total LAL during blood accumulation, confirming the incompatibility of these methods with the lasting survival of the American horseshoe crab. For this reason, new implementations of blood collection–suspension buffer combinations are critical. Here, we evaluated the ability of different blood collection solutions to inhibit exocytosis and subsequently treated the cells with CaCl_2_ to stimulate exocytosis and improve the yield of LAL. Two test methods, chromogenic and turbidimetric tests for LAL activity, were evaluated. Crabs were bled during the bleeding season. The crab blood samples were collected with the following blood collection solutions: citric acid buffer, malic acid buffer, PBS buffer, and PBS–caffeine buffer. The cell pellets were washed with 3% NaCl and subsequently resuspended in LRW or CaCl_2_ to facilitate degranulation. Both the chromogenic test and the turbidimetric assay were used to evaluate the LAL enzyme activity. Citric acid buffer, malic acid buffer, PBS buffer, and PBS–caffeine buffer blocked exocytosis, resulting in the high yields of LAL. There was no observable effect on the activity output of crab size via a chromogenic test with PBS–caffeine buffer during the bleeding season. This protocol substantially benefited prior processes, as the PBS–caffeine collection mixture decreased amoebocyte aggregation/clot formation during processing. Furthermore, we evaluated the specific biochemical parameters of PBS–caffeine-derived LAL. We developed an accessible, promising phosphate–caffeine-based blood collection buffer that prevents amoebocyte degranulation during blood collection, maximizing the LAL yield. Moreover, our analysis revealed that phosphate–caffeine-derived LAL is uniquely adaptable to compatibility with chromogenic and turbidimetric assay techniques. By employing this method for LAL blood extraction, our same-cost approach fostered significantly greater LAL yields, simultaneously ensuring a healthy limulus polyphemus population.

## 1. Introduction

Limulus amoebocyte lysate (LAL) assays are widely used in endotoxin detection for the quality control of biomedical devices and pharmaceutical products [1] and for diagnosing invasive fungal infections [2,3,4,5,6,7]. The LAL test is the most sensitive and reliable method applied for the in vitro detection of bacterial endotoxins [1]. It was introduced in the early 1970s; initially, it provided a more sensitive, semiquantitative means of measuring concentrations of natural environmental endotoxins (NEEs) [8]. In 1972, the LAL test was widely used in the pharmaceutical and medical industries [1]. In 1987, the US FDA issued a guidance document that supported end-product release testing on the sole basis of the LAL test [8].

LAL is an aqueous extract of horseshoe crab (*Limulus polyphemus*) blood cells. The existing LAL method uses horseshoe crab blood to obtain LAL. Three steps are usually used: first, the blood of a horseshoe crab is collected with the collection buffer; then, the blood buffer mixture is centrifuged to obtain the cell pellets, which are subsequently resuspended in the resuspension solution after they are washed. Finally, the cell resuspension mixture was shaken overnight, and LAL was obtained from the supernatant. It usually takes almost one hour from blood collection to resuspend the cell pellets in the resuspension buffer. Armstrong reported that the biochemical analysis of washed blood cells requires that aggregation and degranulation do not occur, which can be accomplished by collecting blood in 0.1 volumes of 2% Tween-20 and 0.5 M LPS-free NaCl, followed by the centrifugation of the cells and washing with 0.5 M NaCl [9]. However, our repeated experiments revealed that most granulates were lost during the blood collection process, making this method not useful for large-scale manufacturing processes. Therefore, many crabs must be used in the manufacturing procedures to obtain enough LAL for commercial purposes if this method is used, which raises concerns about the ecological consequences since horseshoe crabs are limited. Nakamura [10] reported that 50–150 mL of blood per individual was collected in the presence of 3% sodium chloride containing 10 mM caffeine. Amoebocytes were obtained by centrifuging pooled blood and washing twice. The cell pellets were dispersed in the lysis buffer. The suspension was frozen and thawed. The thawed cells were disrupted by homogenization twice. The obtained LAL was used for purification [11]. We performed a similar experiment and reported that most of the granules were not released from the cells after homogenization and were still in the pellet after centrifugation. Therefore, most of the granules were discarded as waste in the pellets, thus reducing the yield of LAL. Notably, this method was developed in the laboratory. This is a longer and more complicated procedure, and the efficiency of the LAL yield is low. Therefore, this approach is also not suitable for large-scale manufacturing.

Additionally, climate change, commercial demand for blood (endotoxin testing), and bait impair the long-term survival of horseshoe crabs [12,13]. The lethal bleeding process harms the population and the ecosystem [14]. Therefore, alternative harvesting strategies are necessary to preserve healthy horseshoe crab populations [15]. Together, these factors necessitate the expansion of alternatives. Feasible alternatives are those with equal efficiency, reducing the number of crabs used in the long run.

Multiple in vitro culture methods and culture media for culturing amoebocyte cells in vitro have been reported [16,17]. However, no such methods have been developed for manufacturing purposes. Recently, recombinant factor C and recombinant factor C, factor B, and preclotting enzyme cascades have been developed for commercial use [18]. However, their price is greater than that of traditional LAL products; moreover, these recombinant products can be used only in chromogenic assays. Recombinant factor G is still under development. Therefore, the traditional LAL test will still be used for a certain amount of time. Therefore, it is still necessary to develop a new LAL method to improve the efficiency of the traditional bleeding method. Solon reported that LPS induces exocytosis in the Limulus GR through the activation of G protein-coupled receptors [19], making it possible to control degranulation during the bleeding process by controlling exocytosis, improving the yield of LAL, and reducing the number of crabs used in the manufacturing process to protect crabs simultaneously.

To prevent premature amoebocyte degranulation during the blood collection process, different approaches have been studied by our group. Carboxylic acid buffers such as citric acid buffers, malic acid buffers, and lactic acid buffers were found to effectively control amoebocyte degranulation and produce high yields of LAL. These LALs work in chromogenic tests. However, they have low activity in turbidimetric assays. Another approach utilized phosphate buffer (pH 6.0) that included divalent cation chelating agents (EDTA and EGTA) as well as glucose (for cellular energy maintenance, as degranulation is an energy-requiring process) as the blood collection buffer to prevent exocytosis during the blood collection process [20]. The cell pellets were washed twice after the blood buffer mixture was centrifuged. Finally, for the ultimate step in the process, the so-called “lysis” or degranulation solution included calcium (5 mM CaCl_2_) to increase the degranulation yield. Calcium is included in the degranulation medium to restore calcium availability, as degranulation (exocytosis) is an intracellular calcium wave-dependent phenomenon [21]. The yield data showed that very high levels of LAL activity could be obtained via this approach. The LAL preparations made in this manner can usually be diluted by 4–8-fold before activity ceases to increase during analytical measurements. We observed a high activity of LAL even after serial dilutions of 4–8 times, and the enzyme activity could still be detected at high endotoxin concentrations, indicating that significant enzymes are retained within the granules during the blood collection process. The LAL activity eventually decreases precipitously with serial dilution; the larger the LAL cascade component content is, the greater the number of serial dilutions before fall-off. Chromogenic and turbidimetric activities were shown to be supported by these LAL preparations.

In addition to the above-described “PBS” method, a second, simpler approach is to utilize a caffeine-rich bleed solution, which also appears to hinder premature degranulation [22]. A bleed solution consisting of 80 mM caffeine in 3% NaCl resulted in degranulation control, and the LAL activity yields were only slightly inferior to those of the PBS-based process. The physiological basis of the blockage of amoebocyte degranulation by caffeine is poorly understood; however, this effect may be related to binding to calcium [23]. Importantly, caffeine-derived LAL works in both chromogenic and turbidimetric assays after 2- to 4-fold dilution.

Since the above-described PBS buffer can result in a high yield of LAL, however, EGTA and EDTA are more expensive than caffeine; moreover, the cell pellets collected from PBS buffer are less aggregated than those collected from the caffeine buffer when they are resuspended in 5 mM CaCl_2_ resuspension buffer and yield slightly greater LAL activity than the caffeine buffer itself. Thus, PBS–caffeine buffer may deserve further development as a blood collection buffer.

This research is the first to develop an optimal blood collection PBS–caffeine buffer after PBS is combined with caffeine. The biochemical characteristics of this PBS–caffeine LAL mixture were determined. Moreover, PBS–caffeine-derived LAL was tested via both chromogenic and turbidimetric assays.

## 2. Results

### 2.1. Evaluation of the Effects of the Use of Citric Acid Buffer, Malic Acid Buffer, PBS Buffer, and PBS–Caffeine Buffer on the Inhibition of Degranulation During Hemolymph Collection Compared with the Effects of the Use of a 3% NaCl Solution

**Figure 1:**
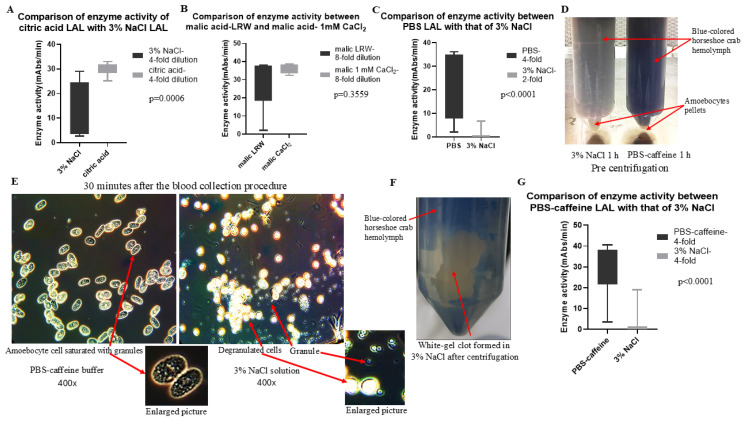
The effects of the use of citric acid buffer, malic acid buffer, PBS buffer, and PBS–caffeine buffer on the inhibition of degranulation during hemolymph collection were evaluated. The blood of the crabs was collected in citric acid buffer, malic acid buffer, PBS buffer, PBS–caffeine buffer, and 3% NaCl solution. Microscopy images of the amoebocyte cells in the blood were taken thirty minutes after collection. After 1 h of incubation at room temperature, the blood–buffer mixture was centrifuged at 1000 rpm (180× *g*) for 5 min. The cell pellets were washed twice with 3% NaCl, resuspended in 5 mM CaCl_2_ resuspension solution, and shaken at 4 °C overnight. The enzyme activity of the supernatants was tested via chromogenic tests, and statistical tests were performed via the GraphPad Prism 10.2 software *t*-test. (**A**) The comparison of the enzyme activity of citric acid LAL with that of 3% NaCl LAL (29.42 ± 2.53 vs. 11.32 ± 11.23 mAbs/min, *p* = 0.0006). (**B**) The comparison of the enzyme activity of malic acid (LAL) resuspended in LAL with that of malic acid (LAL) resuspended in 1 mM CaCl_2_ (29.46 ± 15.35 vs. 36.28 ± 2.66 mAbs/min, *p* = 0.3559). (**C**) The comparison of the enzyme activity of PBS LAL with that of 3% NaCl LAL (23.16 ± 13.44 vs. 0.88 ± 1.85 mAbs/min, *p* < 0.001). (**D**) Blood from horseshoe crabs was collected with 3% NaCl and PBS–caffeine buffer and incubated at room temperature for 1 h. (**E**) The microscopic observation of amoebocytes collected with PBS–caffeine buffer and 3% NaCl solution. (**F**) Gel clot formation in 3% NaCl solution after centrifugation. (**G**) The comparison of the enzyme activity of the PBS–caffeine LAL with that of the 3% NaCl LAL (28.48 ± 12.1 vs. 2.88 ± 6.55 mAbs/min, *p* < 0.001).

Solon reported that citric acid buffer could block the exocytosis of horseshoe amoebocytes in a small-scale experiment [19]. To test whether this buffer also works in a middle-scale experiment, the blood of crabs was collected with citric acid buffer and 3% NaCl as described in the Materials and Methods Section. The cell pellets were resuspended in LRW after being washed twice via 3% NaCl and shaken overnight. The enzyme activity was determined via a chromogenic test after LAL was 4-fold diluted. The results in Figure 1A show that, compared with 3% NaCl, citric acid (LAL) had high activity (29.42 vs. 11.32 mAbs/min, *p* = 0.0006). Because citric acid is a carboxylic acid, we next tested whether another carboxylic acid, malic acid, has a similar function. Following the same procedure as that used for citric acid buffer, malic acid buffer was prepared and tested, and the results in Figure 1B revealed that malic acid (LAL) also had very high enzyme activity via chromogenic tests (malic acid–LRW–LAL 29.46 vs. malic acid–1 mM CaCl_2_–LAL 36.28 mAbs/min, *p* = 0.3559). Unfortunately, both citric acid (LAL) and malic acid (LAL) have low activity in turbidimetric tests, as described below. Since PBS is a very common buffer used in the laboratory, PBS supplemented with EGTA and EDTA was prepared. To our surprise, this PBS buffer also had functions (Figure 1C) similar to those of citric acid buffer and malic acid buffer (the enzyme activity of PBS LAL was 23.16 mAbs/min, whereas the enzyme activity of 3% NaCl LAL was 0.8794 mAbs/min, *p* < 0.0001). Our previous study revealed that caffeine buffer could block exocytosis and produce a high yield of LAL, although the exact mechanism is not known [22]. Our small-scale experiment revealed that the LAL of PBS buffer was slightly greater than that of caffeine buffer. However, the price of EGTA and EDTA is greater than that of caffeine; therefore, PBS–caffeine buffer was further prepared, and its ability to inhibit degranulation during the blood collection process was tested. The blood of eight crabs, including two large, three medium-sized, and three small ones, was collected in PBS–caffeine buffer and 3% NaCl solution (1–1 volume ratio). After the blood collection was finished, 15 µL of the mixed hemolymph mixture was placed on an endotoxin-free glass slide under a cover glass for viewing. The blood buffer mixture was incubated at room temperature for 1 h (Figure 1D) before the mixture was processed as described in the Materials and Methods Section. The activity of the supernatant was tested via a chromogenic assay. Microscopy revealed that degranulation occurred immediately when blood was collected in a 3% NaCl solution. Most of the granules were outside the cells, and few resided inside the cells in the 3% NaCl blood mixture (Figure 1E). Interestingly, most of the granules remained among the granule cells in the PBS–caffeine-collected blood mixture (Figure 1E). A white cloud was observed (Figure 1D), and a glue thread from the top of the tube was observed in a 3% NaCl solution to collect the hemolymph mixture. After centrifugation, the white cloud formed a large gel clot (Figure 1F) in the hemolymph mixture enriched with 3% NaCl. However, no similar phenomenon was observed in the PBS–caffeine buffer-collected hemolymph mixture. The enzyme activity test indicated that almost 90% of the enzyme activity was lost in the 3% NaCl collection-derived enzyme group compared with that in the PBS–caffeine-derived enzyme group (Figure 1G), which was consistent with our findings (Figure 1D) and microscopic observations (Figure 1E). The statistical analysis revealed a significant difference in the enzyme activity yields for LAL derived from PBS–caffeine buffer and LAL derived from 3% sodium chloride (28.48 vs. 2.876 mAbs/min, *p* = 0.0001) (Figure 1G). Therefore, the yield of LAL derived from PBS–caffeine collection buffer significantly exceeded that of traditional methods, indicating that PBS–caffeine buffer could inhibit exocytosis during the blood collection process and that most of the enzyme activity remained in the supernatant of the cell pellet resuspension mixture.

### 2.2. Evaluation of Citric Acid (LAL), Malic Acid (LAL), and PBS LAL in the Turbidimetric Test

The results in Figure 1A–C indicate that citric acid buffers, malic acid buffers, and PBS buffers block the degranulation of amoebocytes during the blood collection procedure and produce high yields of LAL. Next, we evaluated whether these LALs also functioned in turbidimetric assays. These LALs were prepared as described in the Materials and Methods Section after they were incubated at 4 °C for three weeks. The enzyme activity of these LALs was similarly adjusted via LRW. Then, they were formulated and lyophilized as described in the Methods Section. The lyophilized powder was dissolved via 0.2 M Tris-Cl buffer (pH 7.4). The turbidimetric activity of these LALs was tested following the procedure described in the Methods Section. As shown in Table 1, PBS LAL had better activity in the turbidimetric test; however, citric acid LAL and malic acid LAL had lower activity in the turbidimetric test, although their chromogenic enzyme activities were similar. Therefore, the use of PBS buffer deserves further study. Caffeine buffers can block exocytosis, and caffeine is less expensive than EGTA and EDTA. PBS–caffeine buffer was chosen for further study as described below.

### 2.3. CaCl_2_ (5 mM) Improved the Yield of LAL

**Figure 2:**
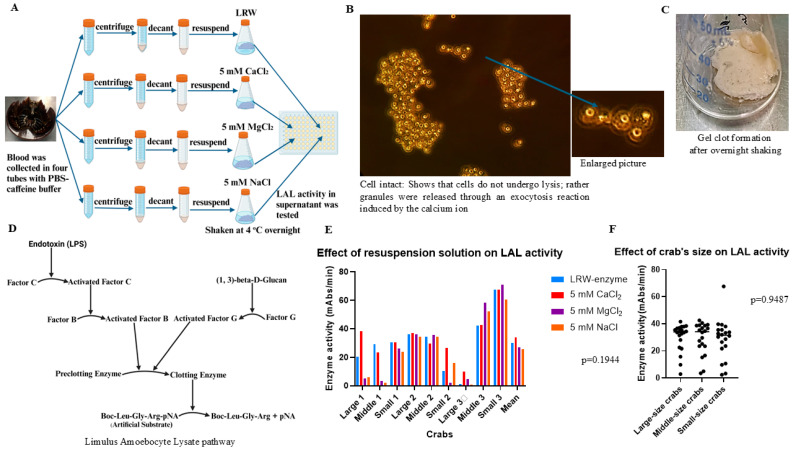
CaCl_2_ (5 mM) improved the yield of LAL. For each crab, 15 mL of blood was collected in four Corning tubes from 60 mL of total blood drawn from each crab. These four Corning tubes were prefilled with 15 mL of PBS–caffeine buffer prior to the collection of blood from each crab. The blood–buffer mixture was centrifuged at 1000 rpm for 5 min. The cell pellets were washed twice with 3% NaCl solution and resuspended in LRW, 5 mM CaCl_2_, 5 mM MgCl_2_, or 5 mM NaCl. After shaking at 4 °C overnight, we tested the enzyme activity via the chromogenic method. One-way ANOVA was conducted via GraphPad Prism 10.2 software to compare the enzyme activity. (**A**) Experimental procedure. (**B**) Microscopy image after the cell pellet was resuspended overnight. (**C**) Gel clot formation after shaking overnight. (**D**) Limulus amoebocyte lysate pathway. (**E**) The comparison of enzyme activity in the supernatant when the cell pellets were resuspended in LRW (30.1 ± 19.15), 5 mM CaCl_2_ (33.88 ± 15.9), 5 mM MgCl_2_ (26.91 ± 25.52), or 5 mM NaCl (25.54 ± 21.59) mAbs/min overnight, *p* = 0.1944. (**F**) The comparison of LAL activity in different sizes of crabs. The average enzyme activities of large crabs, mid-sized crabs, and small crabs were 29.99 ± 10.32, 29.22 ± 11.62, and 28.74 ± 14.71 mAbs/min, respectively (*p* = 0.9487).

To test which the resuspension solution could maximize the yield of LAL, the blood of nine crabs, including three “large” crabs (20 cm), three “mid-sized” crabs (15 cm), and three “small” crabs (8 cm), was collected in PBS–caffeine buffer. Blood was collected from each crab in four 50 mL Corning tubes containing 15 mL of PBS–caffeine buffer. In each tube, 15 mL of blood was collected. The total volume of the blood and buffer mixture was approximately 30 mL. The mixture was then processed as described in the Materials and Methods Section. The cell pellets were subsequently resuspended in LRW, 5.0 mM CaCl_2_, 5.0 mM MgCl_2_, or 5.0 mM NaCl (Figure 2A). The enzyme activity of the supernatant was tested via the chromogenic test method. Microscopy revealed that most of the cells remained intact. All the granules inside the cells disappeared (Figure 2B), and a gel clot formed after overnight shaking (Figure 2C). The limulus amoebocyte lysate cascade enzyme reaction pathway is shown in Figure 2D. The enzyme activity varied among the different crabs, as shown in Figure 2E. For example, the maximal enzyme activity in middle-sized crab-1 occurs in LRW; however, this activity emerges in the presence of 5.0 mM CaCl_2_ in large crab-1. On average, the maximal enzyme activity was produced when the cell pellets were resuspended in 5 mM CaCl_2_ (Figure 2E). However, the statistical analysis revealed that there was no significant difference in the average enzyme activity among the nine crab species when the cell pellets were resuspended in the following four different solutions: LRW, 5.0 mM CaCl_2,_ 5.0 mM MgCl_2,_ and 5.0 mM NaCl (30.1, 33.88, 26.9, and 25.54 mAbs/min, respectively, *p* = 0.1944) (Figure 2E). This result is similar to that obtained with caffeine buffer [22]. Notably, crab size did not seem to have an observable effect on the activity of LAL via chromogenic tests (Figure 2F).

### 2.4. Effects of pH and Temperature on Enzyme Activity

**Figure 3:**
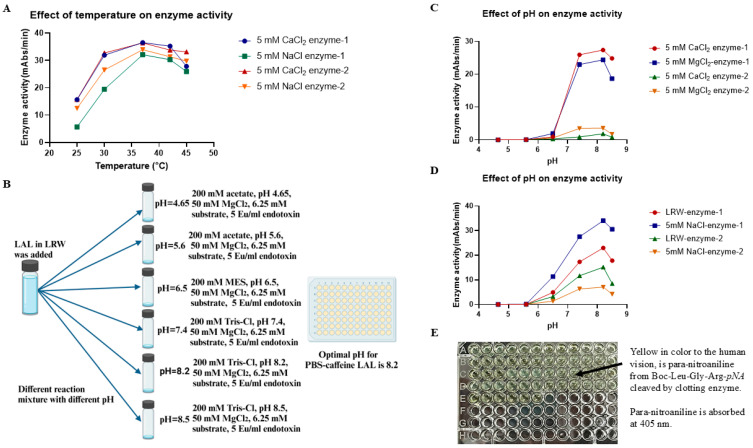
The effects of temperature and pH on enzyme activity. (**A**) The effects of temperature on enzyme activity. Fifty microliters of PBS–caffeine LAL was incubated with 50 µL of the reaction mixture (140 mM Tris-Cl, pH 7.4, 50 mM MgCl_2_, 6.25 mM substrate, 5 Eu/mL endotoxin) in four 96-well plates. The plates were put into four plate readers set at 25 °C, 30 °C, 37 °C, and 42 °C. The reaction was monitored for 1 h. (**B**) Experimental procedure for determining the effects of pH on enzyme activity. Fifty microliters of PBS–caffeine-treated LAL were incubated with 10 µL of buffer at six different pH values (200 mM acetate, pH 4.65, 200 mM acetate, pH 5.6, 200 mM MES, pH 6.5, 200 mM Tris-Cl, pH 7.4, 200 mM Tris-Cl, pH 8.2, and 200 mM Tris-Cl, pH 8.5) in a 96-well plate at room temperature for 5 min. Then, 40 µL of each reaction mixture (50 mM MgCl_2_, 6.25 mM substrate, and 5 Eu/mL endotoxin) was added, and the plate was subsequently placed in a plate reader at 37 °C. The reaction was monitored for 1 h. (**C**,**D**) The effects of pH on enzyme activity. Eight different kinds of PBS–caffeine LAL were incubated with buffers at six different pH values as described in (**B**). The reaction mixture was added, and the plate was subsequently placed in a plate reader at 37 °C. The reaction was monitored for 1 h. (**E**) The reaction was performed on a 96-well pyro plate. The color of the reaction mixture turned yellow 1 h after the reaction when para-nitroaniline from Boc-Leu-Gly-Arg-*pNA* was released by clotting enzyme cleavage.

To test the effect of temperature on enzyme activity, 50 µL of the PBS–caffeine enzyme mixture (Figure 3A) was incubated with 50 µL of the reaction mixture in four 96-well plates as described in the Materials and Methods Section. The plates were placed into four plate readers set at 25 °C, 30 °C, 37 °C, and 42 °C. The chromogenic assay was monitored for 1 h. Our analysis revealed that enzyme activity was not apparent when the reaction temperature was 25 °C. The enzyme activity gradually increased as the reaction temperature increased from 25 °C to 37 °C. Additionally, the enzyme activity peaked at 37 °C and decreased gradually when the temperature increased to 42 °C (Figure 3A), indicating that the optimal temperature for this cascade enzyme reaction was 37 °C.

Next, we tested the effect of pH on the activity of the PBS–caffeine enzymes. Fifty microliters of PBS–caffeine enzyme were incubated with 10 µL of buffer at pH values of 4.65, 5.6, 6.5, 7.4, 8.2, and 8.5 in a 96-well plate at room temperature for 5 min, after which 40 µL of each reaction mixture was added as described in the Materials and Methods Section. The plate was subsequently placed in a plate reader at a reaction temperature of 37 °C. The chromogenic assay mixture was monitored at 405 nm for 1 h (Figure 3B). The results revealed that no enzyme activity was detected when the pH of the reaction mixture was less than 5.6. The enzyme activity gradually increased when the pH of the reaction mixture increased from 6.5, and the maximal enzyme activity was obtained when the pH of the reaction mixture was 8.2. The enzyme activity decreased when the pH increased from 8.2 to 8.5 (Figure 3C,D). Therefore, the optimal pH for the PBS–caffeine cascade enzyme is 8.2. Notably, these results are also similar to those of caffeine LAL [22], indicating that caffeine LAL and PBS–caffeine LAL have similar pH and temperature characteristics. Notably, the reaction mixture turned yellow in color because para-nitroaniline from Boc-Leu-Gly-Arg-*pNA* was released by clotting enzyme cleavage (Figure 3E).

### 2.5. Effect of Ions on Enzyme Activity

**Figure 4:**
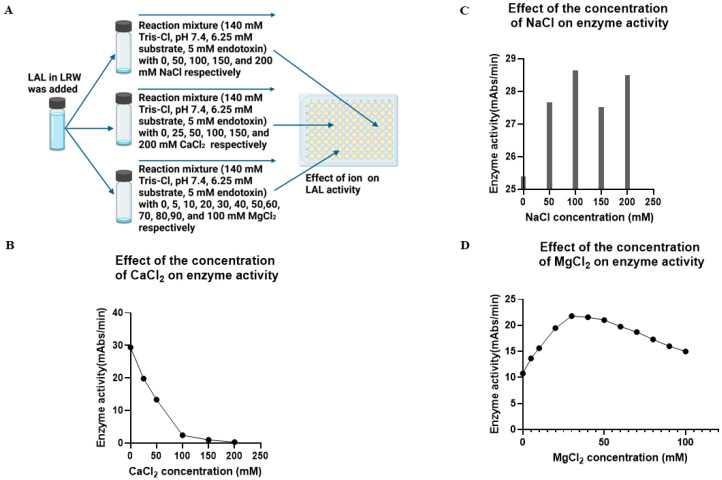
The effect of ions on enzyme activity. (**A**) Experimental procedure. (**B**) The effect of CaCl_2_ on enzyme activity. The PBS–caffeine enzyme mixture was incubated with 0, 25, 50, 100, 150, or 200 mM CaCl_2_ in 96-well plates at room temperature for 5 min, after which 50 µL of the reaction mixture (140 mM Tris-Cl, pH 7.4, 6.25 mM substrate, and 5 Eu/mL endotoxin) was added. The plate was placed in a plate reader, and the reaction was monitored at 37 °C for 1 h. (**C**) The effect of NaCl on enzyme activity. The PBS–caffeine enzyme mixture was incubated with 0, 50, 100, 150, or 200 mM NaCl in 96-well plates at room temperature for 5 min, after which 50 µL of the reaction mixture (140 mM Tris-Cl, pH 7.4, 6.25 mM substrate, and 5 Eu/mL endotoxin) was added. The plate was placed in a plate reader, and the reaction was monitored at 37 °C for 1 h. (**D**) The effect of MgCl_2_ on enzyme activity. The PBS–caffeine enzyme mixture was incubated with 0, 5, 10, 20, 30, 40, 50, 60, 70, 80, 90, or 100 mM MgCl_2_ in 96-well plates at room temperature for 5 min, after which 50 µL of the reaction mixture (140 mM Tris-Cl, pH 7.4, 6.25 mM substrate, and 5 Eu/mL endotoxin) was added. The plate was placed in a plate reader, and the reaction was monitored at 37 °C for 1 h.

To test the effect of ions on enzyme activity, the PBS–caffeine enzyme mixture was incubated with different concentrations of NaCl, CaCl_2_, and MgCl_2_ in 96-well plates at room temperature for 5 min, after which 50 µL of the reaction mixture was added as described in the Materials and Methods Section (Figure 4A). The plate was placed in a plate reader, and the chromogenic reaction was monitored at 37 °C for 1 h. These results indicated that CaCl_2_ inhibited enzyme activity. When the concentration of CaCl_2_ was 50 mM, 50% of the enzyme activity was blocked. Almost no enzyme activity was detected when the concentration of CaCl_2_ was 200 mM (Figure 4B). In contrast, NaCl did not inhibit the enzyme reaction at this concentration (Figure 4C). Notably, the inhibitory effect increased with increasing concentrations of CaCl_2_ (Figure 4B). However, when the concentration of CaCl_2_ was less than 10 mM, its inhibitory effect was limited. Importantly, 5 mM CaCl_2_ was used to stimulate exocytosis during the resuspension step of the bleeding process. However, when this LAL was formulated in the endotoxin test kit, the concentration of CaCl_2_ further decreased. Therefore, its inhibitory effect on the endotoxin test may be negligible. On the other hand, the enzyme activity gradually increased as the concentration of MgCl_2_ increased from 5 to 30 mM and then decreased from 30 to 100 mM (Figure 4D). Thus, the optimal Mg^2+^ concentration for this PBS–caffeine cascade enzyme was 30 mM. Interestingly, partial enzyme activity was detected when no MgCl_2_ was added to the reaction mixture, possibly because Mg^2+^ from the cell contributed to this biochemical reaction.

### 2.6. CaCl_2_ Inhibited the Effect of PBS–Caffeine Buffer on Exocytosis

**Figure 5:**
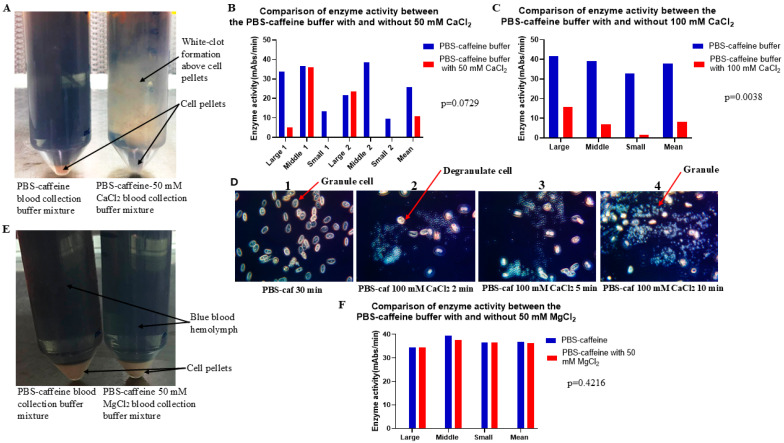
CaCl_2_ inhibited the effect of the PBS–caffeine buffer on exocytosis. Blood from two large, two middle-sized, and two small crabs was collected with PBS–caffeine buffer or PBS–caffeine buffer supplemented with 50 mM CaCl_2_. The blood buffer mixture was processed as described in the Materials and Methods Section. The cell pellets were resuspended in 5 mM CaCl_2_ resuspension solution, and the mixture was shaken at 4 °C overnight. The enzyme activity of the supernatant was tested via the chromogenic method, and the statistical test was performed via the GraphPad Prism 10.2 software *t*-test. The same procedure was used for the other two experiments: PBS–caffeine buffer and PBS–caffeine buffer with 100 mM CaCl_2_ or PBS–caffeine buffer and PBS–caffeine buffer with 50 mM MgCl_2_. (**A**) Blood was collected in PBS–caffeine buffer or PBS–caffeine buffer supplemented with 50 mM CaCl_2_. One hour after blood collection, the blood buffer mixture was centrifuged at 1000 rpm (180× *g*) at 10 °C for 5 min. (**B**) The comparison of enzyme activity when blood was collected in PBS–caffeine buffer or PBS–caffeine buffer supplemented with 50 mM CaCl_2_ (25.68 ± 12.47 vs. 10.93 ± 15.32 mAbs/min, *p* = 0.0729). (**C**) The comparison of enzyme activity when blood was collected in PBS–caffeine buffer or PBS–caffeine buffer supplemented with 100 mM CaCl_2_ (37.88 ± 4.544 vs. 8.171 ± 7.179 mAbs/min, *p* = 0.0038). (**D**) The microscopic observation of the amoebocyte degranulation process. As shown in the picture, more granules were increasingly released with increasing time in the presence of 100 mM CaCl_2_. (**E**) Blood was collected in PBS–caffeine buffer or PBS–caffeine buffer with 50 mM MgCl_2_. One hour later, the blood buffer mixture was centrifuged at 1000 rpm (180× *g*) at 10 °C for 5 min. (**F**) The comparison of enzyme activity when blood was collected in PBS–caffeine buffer or PBS–caffeine buffer supplemented with 50 mM MgCl_2_ (36.81 ± 2.47 vs. 36.2 ± 1.61 mAbs/min, *p* = 0.4216).

Until recently, the mechanism by which PBS–caffeine buffer affects exocytosis was poorly understood. Our previous study indicated that the caffeine buffer-mediated inhibition of exocytosis may involve Ca^2+^. To further test whether CaCl_2_ could block the effect of PBS–caffeine buffer on degranulation during the blood collection process, the blood of two large crabs, two medium crabs, and two small crabs was collected in PBS–caffeine buffer or PBS–caffeine buffer supplemented with 50 mM CaCl_2_. The blood buffer mixture was treated as described in the Materials and Methods Section, and the cell pellets were resuspended in 5 mM CaCl_2_ resuspension solution. The enzyme activity of the supernatant was tested via the chromogenic method. Figure 5A shows that a white turbid gel formed in the mixture of crab hemolymph with PBS–caffeine buffer containing 50 mM CaCl_2_ (Figure 5A, right side tube); however, no similar phenomenon was observed in the mixture of crab hemolymph with PBS–caffeine buffer (Figure 5A, left side tube). The enzyme activity test revealed that most of the enzyme activity was lost when the blood was collected in PBS–caffeine buffer supplemented with 50 mM CaCl_2_ (Figure 5B, orange column). On the other hand, high enzyme activity was still detected in the supernatant when the blood was collected in PBS–caffeine buffer (25.68 and 10.93 mAbs/min, *p* = 0.0729) (Figure 5B, blue column). The concentration of CaCl_2_ was increased from 50 to 100 mM. An increasing amount of enzyme activity was lost (Figure 5C). Statistical analysis revealed that there was a significant difference between these two groups (37.88 and 8.171 mAbs/min, *p* = 0.0038) (Figure 5C). To further confirm this observation, 10 µL of a PBS–caffeine buffer blood mixture was mixed with a final concentration of 100 mM CaCl_2_; the degranulation process was observed under a microscope, as shown in Figure 5D. The degranulation process started when the blood buffer mixture was mixed with CaCl_2_ for approximately 2 min (Figure 5D2), and as time progressed, degranulation increased in parallel. Notably, almost all the granules resuspended in CaCl_2_ were released at 10 min (Figure 5D4). On the other hand, most of the granules remained inside the amoebocyte in PBS–caffeine buffer even after 30 min (Figure 5D1).

In contrast, 50 mM MgCl_2_ did not inhibit the effect of PBS–caffeine buffer on exocytosis during the blood collection process (Figure 5E). The enzyme activities of PBS–caffeine buffer and PBS–caffeine buffer with 50 mM MgCl_2_ were very similar (36.81 and 36.2 mAbs/min, *p* = 0.4216) (Figure 5F), suggesting that PBS–caffeine buffer inhibits exocytosis during the blood collection process through calcium ion interference. However, further experiments should be conducted to elucidate the exact mechanism by which PBS–caffeine buffer affects exocytosis.

### 2.7. PBS–Caffeine LAL Functioned in the Turbidimetric Assay

**Figure 6:**
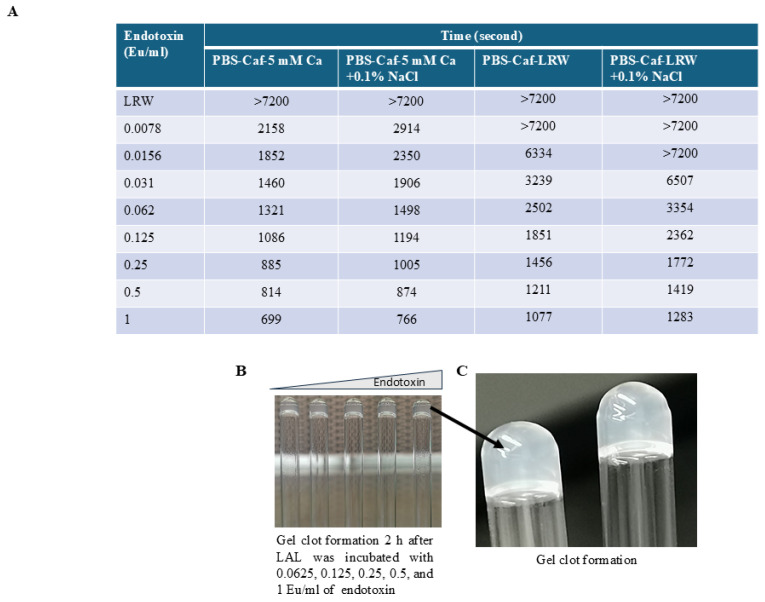
PBS–caffeine LAL functioned in the turbidimetric assay. Six milliliters of PBS–caffeine LAL was incubated with 240 µL of 1 M MgCl_2_ for 10 min, after which the mixture was divided into two parts (3 mL each). To one part of the LAL mixture, 100 µL of 30% NaCl was added; to the other part, 100 µL of LAL reagent water was added. After being shaken for 10 min, the mixture was lyophilized at −50 °C. The enzyme activity was tested via the turbidimetric method via a PKFLEX tube reader. (**A**) The onset time of the PBS–caffeine enzyme with or without NaCl at different concentrations of endotoxin; (**B**,**C**) Gels were formed 2 h after LAL was incubated with 0.0625, 0.125, 0.25, 0.5, or 1 Eu/mL endotoxin.

To test whether the LAL of PBS–caffeine works in turbidimetric assays and to determine the effect of NaCl on turbidimetric parameters, a PBS–caffeine LAL was prepared as described in the Materials and Methods Section. The prepared mixture was lyophilized at −50 °C. A white powder formed after 48 h of lyophilization. LAL reagent water (LRW) (2.5 mL) was added to each vial. The powder was easily dissolved. The enzyme activity was tested via 2-fold serial dilutions of endotoxin (from 1 Eu/mL to 0.0078 Eu/mL) via a turbidimetric method with a PKFLEX tube reader as described in the Materials and Methods Section. The results in Figure 6A indicate that the onset time gradually decreased as the endotoxin concentration increased. Notably, a gel formed tightly when the endotoxin concentration was greater than 0.062 Eu/mL (Figure 6B,C). These turbidimetric assay results demonstrated that the PBS–caffeine LAL works in turbidimetric assays. Interestingly, NaCl seems to inhibit the turbidimetric reaction such that the onset time becomes longer (Figure 6A).

## 3. Discussion

This study, which has been almost three years long, is the first to report that PBS–caffeine blood collection buffer could inhibit exocytosis during the blood collection process of horseshoe crabs, increasing the yield of LAL, reducing the number of horseshoe crabs used during the LAL manufacturing procedure by at least 50% to 75%, and protecting the crabs in the long term.

It has been more than five decades since the discovery that horseshoe crab blood could be used for natural environment endotoxin tests [8]. LAL is an aqueous extract of horseshoe crab (Limulus polyphemus) blood cells [1]. The gel clot LAL test was approved by the Food and Drug Administration (FDA) in the 1970s and has been widely adopted as the official method for detecting bacterial endotoxins [24]. Since then, the LAL test has been extensively evaluated as an extremely sensitive, specific, simple, rapid, and economical method for detecting endotoxins. Various alternative techniques have also been developed without the use of LAL technology since the 1970s [1]. Endotoxin assays can be generally divided into two categories: LAL and non-LAL assays. The LAL assay is officially used with a different type of formulation that comprises conventional or endotoxin-specific reagents for both endpoint and kinetic assay formats [25,26]. Other techniques (modified LAL) include ESP, the bioluminescence assay using mutant luciferase, and the ELISA-like assay [1]. As a different approach, the Lab-On-a-Chip Application Development Portable Test System (LOCAD-PTS) was introduced as a modified technique to further improve the usability and simplicity of the LAL assay [27]. Cell-based endotoxin assays have been developed using different immune cells and cell lines, such as human neutrophils, monocytes, and human embryonic kidney (HEK) 293 cells. An alternative in vitro pyrogen test, the monocyte activation test (MAT), was developed to detect non-endotoxin pyrogen (NEP) as well as endotoxin.

On the basis of a different mechanism, the endotoxin activity assay (EAA) measures the production of reactive oxygen species by human neutrophils from whole-blood samples, followed by the formation of LPS–anti-LPS–antibody complexes. Both MAT and EAA have low or limited specificity against endotoxins because of the mechanism by which the analytes are generated during a series of cellular responses [1].

Several other methodologies known as indirect endotoxin assays are available. These include measurements of serum lipoproteins, anti-endotoxin antibodies, and LPS-binding protein (LBP). However, no correlation was found between endotoxemia and LBP levels [1].

The LAL assay has established a firm position as an alternative to the rabbit pyrogen test; thus, the horseshoe crab has already proven to be an extremely beneficial organism for biomedical use. However, there is growing awareness of the importance of protecting endangered species; thus, different approaches have been used to protect these carbs. One approach involves the use of recombinant technology, and the other approach involves improving the yield of LAL during the bleeding process and reducing the number of crabs used in the bleeding procedure.

Different methods are used to collect blood from horseshoes [28]. Nakamura [10] reported the use of homogenization twice to disrupt granulated cells to obtain LAL. A similar experiment was carried out by our group. Many granule cells were not broken, and most of the granules were not released from the cells after homogenization. Therefore, these granules were discarded as waste in the pellets after centrifugation, thus reducing the yield of LAL. Our observations revealed that granule loss occurred primarily in the blood collection step of the bleeding process when 3% NaCl solution was used (Figure 1). Thus, reducing premature granule loss during the blood collection step and enhancing granule exocytosis in the resuspension step present opportunities for improving the LAL activity yield. Therefore, the ability of new bleeding solutions designed to prevent degranulation during the blood collection step and enhance degranulation in the resuspension step was possible on the basis of previous research on *Limulus* amoebocytes.

Various approaches have been investigated. Citric acid buffer (3% NaCl, 100 mM glucose, 25 mM citric acid, 30 mM citrate, and 10 mM EDTA, pH 4.6) was found to prevent degranulation during the blood collection process [19,29] and to result in a high yield of citric acid LAL (Figure 1A). Both citric acid and malic acid are carboxylic acids. Citric acid [HOOC-CHOH (CH2COOH)_2_] has three carboxylic groups, and malic acid (HOOC-CHOH-CH2COOH) has two carboxylic groups. The calcium chelation strength increases with increasing carboxylate groups [30]. Malic acid blood collection buffers were also prepared using the same formulation as the citric acid buffer. Malic acid blood collection buffers were found to prevent degranulation during the blood collection process, resulting in a high yield of LAL (Figure 1B). Unfortunately, although these LALs have high enzyme activities in the chromogenic assay (Figure 1A,B), they seemed to lose their activities in the turbidimetric assay (Table 1).

Through pH adjustment of these buffers, we found that if the pH of these buffers was greater than 5.6, LAL activity could be retained in the turbidimetric assay. Unfortunately, both the citric acid buffer and the malic acid buffer almost completely lost their buffering capacity when the pH exceeded 5.6. Accordingly, for additional research, PBS buffers, which have a strong buffering capacity at pH 5.6, were chosen for further experiments; 3% NaCl was used to maintain osmotic pressure; and 100 mM glucose was used to provide energy for cell metabolic processes, including exocytosis.

Evaluating 20 to 100 mM PBS suggested that 50 mM PBS was a better solution. This PBS buffer also functions in both chromogenic and turbidimetric assays. This is an important observation because, while chromogenic activity requires only that the protease zymogens of the cascade remain functional, turbidimetric activity requires both a functional cascade and a functional coagulogen. Maintaining the latter function is not trivial, as coagulogen contains 16 cysteines that participate in 8 disulfide linkages [31,32]. A reduction in these disulfide bonds may result in “scrambling” of the coagulogen structure after the reoxidation of the disulfides or the simple loss of appropriate clotting enzyme substrate cleavage sites. The low pH (reducing conditions) of the carboxylic acid-based buffers (malic and citric acids) may be responsible for the inactivation of the turbidimetric capability of the LAL prepared with those compounds.

Caffeine-containing buffers could also prevent degranulation, although the mechanism is unknown [22]. Murer reported that caffeine at a concentration of 10–30 mM prevents the clumping and disintegration of amoebocytes. The disruption of caffeine-treated cells subsequently resulted in the activation of the coagulation system, resulting in clotting (Figure 1F). Figure 5 shows that treatment with PBS–caffeine buffer supplemented with 50 mM calcium chloride blocked the function of the PBS–caffeine buffer. As PBS also binds Ca^2+^, PBS–caffeine buffer requires a high concentration of Ca^2+^ to inhibit PBS–caffeine buffer function (Figure 5B,C). As shown in Figure 5D, when the concentration of Ca^2+^ was 100 mM, degranulation occurred; however, the cells did not clump clearly (Figure 5D). Notably, Mg^2+^ does not have this effect under similar conditions (Figure 5E,F). Therefore, PBS–caffeine may block exocytosis by binding to Ca^2+^.

Our small-scale experiments indicated that caffeine buffers produced a slightly lower yield of LAL activity than did PBS [22]. Notably, after the cell pellets were resuspended in 5.0 mM CaCl_2_, the caffeine buffer cell pellets were easily attached to the walls of Corning tubes; this resuspension phenomenon made it difficult to pool the aliquots into an Erlenmeyer flask and reduce the yield of LAL. No similar phenomenon was observed with PBS buffer. However, caffeine is much less expensive than EGTA or EDTA are, making it appropriate for further investigation. Accordingly, the ability of the PBS buffer components to reduce clot formation in caffeine bleed solution was investigated.

During the bleeding season, each component of PBS was individually added to an 80 mM caffeine mixture to check for clot formation and the yield of LAL. The results indicated that adding 50 mM phosphate (pH 6.0) to the caffeine solution reduced cell clot formation and improved the yield of LAL [22]. Blood was further collected from 60 crabs in PBS–caffeine buffer. The results showed that the PBS–caffeine buffer produced both high activity and a high yield of LAL. Notably, the PBS–caffeine LAL works well in chromogenic and turbidimetric assays. Importantly, the degranulation process is an exocytosis process, not a cell lysis process. Most cells remained intact after degranulation (Figure 2B). Moreover, this PBS–caffeine method is a highly efficient and easy operation method that is suitable for manufacturing. Almost all the granules were released after stimulation with Ca^2+^ (Figure 2B).

LAL alternatives based on recombinant technologies have recently attracted much attention from the perspective of the global pharmacopoeia [33,34,35]. Recombinant alternatives are specific to endotoxin and consist of two types of reagents: recombinant factor C and recombinant factor C, recombinant factor B, and the preclotting enzyme cascade. Recently, Bolden et al. reviewed the currently available recombinant alternatives to horseshoe crab blood lysates and their comparability [18]. However, recombinant reagents can only be used for chromogenic tests. Dubczak compared two limulus amoebocyte lysates (LALs) (kinetic chromogenic LALs from Charles River (KCA) and kinetic chromogenic LALs from Lonza (Kinetic-QCL^TM^)) with three commercially available recombinant factors (rFCs) [Rfc from Lonza (PyroGene^TM^), Rfc from bioMérieux (EndoZyme^®^ II), and Rfc (Endozyme^®^ II go) from bioMérieux]. His study also includes a recombinant reagent that has been developed to include all three of the enzymes involved in the LAL coagulation cascade. A statistical analysis of 128 samples containing environmental endotoxins revealed that, at the 5% level of significance, noninferiority between the two traditional methods was achieved. However, the noninferiority claim could not be made with any of the recombinant reagents. Notably, very low concentrations of endotoxin cannot be detected by recombinant reagents; however, it can be detected by conventional LAL reagents [8]. Notably, recombinant factor G is currently under development.

In summary, the PBS–caffeine bleeding method increased the yield of LAL and reduced the number of crabs used. It is an easier, rapid method. The manufacturer could use the current equipment to update the bleeding method and reduce the cost of purchasing the new instrument. Moreover, the PBS–caffeine LAL could be used in both chromogenic assays and turbidimetric tests. The customers using gel clots and turbidimetric tests do not have to worry about changing their equipment for the endotoxin test, which also reduces their costs.

## 4. Materials and Methods

### 4.1. Amoebocyte Collection Solution

The collection solution formulations that were assessed are as follows: citric acid buffer (26 mM citric acid, 30 mM sodium citrate, 10 mM EDTA, 100 mM glucose, and 3% NaCl, pH 4.6), malic acid collection buffer (3% NaCl, 100 mM glucose, 26 mM malic acid, 30 mM malic acid salt, and 10 mM EDTA, pH 4.0), PBS buffer (3% NaCl, 100 mM glucose, 50 mM KH_2_PO_4_, 20 mM EGTA, and 10 mM EDTA, pH 5.6), and PBS–caffeine buffer (25 mM KH_2_PO_4_, 25 mM Na_2_HPO_4,_ 100 mM glucose, 3% NaCl, and 80 mM caffeine, pH 6.0). The cell-washing formulation that was tested is as follows: 3% NaCl solution. The cell resuspension/degranulation-stage solutions that were evaluated are as follows: LAL reagent water (LRW), 1 mM CaCl_2_, 5 mM CaCl_2_, 5 mM MgCl_2,_, and 5 mM NaCl. The blood of American horseshoe crabs was provided by the manufacturer of the LAL reagent. No ethics approval was needed for this experiment.

### 4.2. Evaluation of the Effects of the Use of Different Blood Collection Solutions on the Inhibition of Degranulation During Hemolymph Collection

To evaluate the effects of different blood collection buffers on the inhibition of degranulation during hemolymph collection, 25 mL of hemolymph was collected with an equal volume of blood collection buffer from each crab. To compare the effects of different blood collection buffers on the inhibition of degranulation, 50 mL of hemolymph was collected from each crab with both solutions. After the blood buffer mixture was incubated at room temperature for 1 h, it was centrifuged at 10 °C and 1000 rpm (180× *g*) for 5 min, the supernatant was removed, and 25 mL of washing buffer was added. After centrifugation at 10 °C and 1000 rpm (180× *g*) for 5 min, the supernatant was decanted, 25 mL of washing buffer was added, and the mixture was centrifuged again at 10 °C and 1000 rpm (180× *g*) for 5 min. The supernatant was subsequently removed, and the resuspension buffer was added at a ratio of 1:8 (1 g of pellet was added to 8 mL of buffer). The mixture was vortexed for 30 s. The mixture was subsequently decanted into a 50 mL Erlenmeyer glass flask and shaken at 100 rpm overnight at 4 °C. After the mixture was shaken overnight, the gel clot precipitated at the bottom. The supernatant was transferred to a sterile glass tube for storage. Moreover, the enzyme activity was tested via the chromogenic method. Notably, the 3% NaCl solution was used as a control buffer. To compare the effects of citric acid buffer with 3% NaCl on the inhibition of degranulation during the blood collection process, the blood of 8 crabs was collected with both citric acid buffer and 3% NaCl following the procedure described above, and the enzyme activity was tested via the chromogenic method. The average enzyme activity was calculated, and the statistical test was performed via the GraphPad Prism 10.2 software *t*-test. To compare the effects of 1 mM CaCl_2_ with those of LAL reagent water (LRW) on exocytosis in the resuspension step, the blood of 5 crabs was collected with malic acid buffer following the procedure described above. Each crab’s blood sample was collected in two tubes containing an equal volume of malic acid buffer: one tube of cell pellets was resuspended in LRW, and the other tube was resuspended in a 1 mM CaCl_2_ solution (8 mL of resuspension solution was added per gram of pellet). Enzyme activity was tested via the chromogenic method. The average enzyme activity was calculated, and the statistical test was performed via the GraphPad Prism 10.2 software *t*-test. To compare the effects of PBS buffer with 3% NaCl on the inhibition of degranulation during the blood collection process, the blood of 12 crabs was collected with both PBS buffer and 3% NaCl following the procedure described above, and the enzyme activity was tested via the chromogenic method. The average enzyme activity was calculated, and the statistical test was performed via the GraphPad Prism 10.2 software *t*-test. To compare the effects of PBS–caffeine buffer with 3% NaCl on the inhibition of degranulation during the blood collection process, the blood of 8 crabs was collected with both PBS–caffeine buffer and 3% NaCl following the procedure described above, and the enzyme activity was tested via the chromogenic method. The average enzyme activity was calculated, and the statistical test was performed via the GraphPad Prism 10.2 software *t*-test.

### 4.3. Microscopic Examination of the Hemocytes

After the blood was collected with PBS–caffeine buffer and 3% NaCl, 15 µL of the mixed hemolymph mixture was placed on an endotoxin (LPS)-free glass slide, and then, LPS-free cover glasses were used to cover a drop of the mixture. This glass slide was immediately placed under a microscope to observe the granulocytes (10 × 40). To observe the effects of CaCl_2_ on exocytosis, 100 mM CaCl_2_ was mixed with a PBS–caffeine hemolymph solution on an LPS-free glass slide, the glass slide was covered with an LPS-free glass cover, the degranulation process was observed via microscopy, and images were taken with a camera connected to the microscope at different time intervals.

### 4.4. Chromogenic Methods

The activity analysis was performed in 96-well plates. LAL was 2-fold serially diluted from 1:2, 1:4, and 1:8 to 1:16 with LRW (LAL Reagent Water), 50 µL of LAL was added to the wells in triplicate, and then 50 µL of the reaction mixture was added (0.14 M Tris, pH 7.4, 50 mM MgCl_2,_ 6.25 mM chromogenic peptide, and 5 Eu/mL endotoxin). The plate was placed into a BioTek ELx808 plate reader, and the absorbance was read at 405 nm. Each sample was tested in triplicate, and the reaction was monitored at 37 °C for 60 min, after which the average Vmean with a coefficient of variation (CV) of test values of < 20% per requirement was analyzed and used to compare the enzyme activity in different reaction mixtures. At the end of the reaction, the reaction mixture turned yellow because para-nitroaniline from Boc-Leu-Gly-Arg-*pNA* was released by clotting enzyme cleavage.

### 4.5. Evaluation of the Effect of the Resuspension Solution on the Yield of LAL

To test which resuspension solution could maximize the yield of LAL, the blood of nine crabs was collected in PBS–caffeine buffer. Blood was collected from each crab in four 50 mL Corning tubes containing 15 mL of PBS–caffeine buffer. In each tube, 15 mL of blood was collected. The total volume of the blood and buffer mixture was approximately 30 mL. Next, the blood buffer mixture was incubated at room temperature for 30 min; the mixture was centrifuged at 10 °C and 1000 rpm (180× *g*) for 5 min, the supernatant was removed, and 25 mL of washing buffer was added. After centrifugation at 10 °C and 1000 rpm (180× *g*) for 5 min, the supernatant was decanted, 25 mL of washing buffer was added, and the mixture was centrifuged again at 10 °C and 1000 rpm (180× *g*) for 5 min. The supernatant was removed. The cell pellets were subsequently resuspended in LRW, 5.0 mM CaCl_2_, 5.0 mM MgCl_2_, or 5.0 mM NaCl in a ratio of 1:8 (1 g of pellet was added to 8 mL of buffer). After vortexing for 30 s, the cell pellet mixture was decanted into a 50 mL glass flask, which was shaken at 4 °C overnight. The enzyme activity of the supernatant was tested via the chromogenic test method. The average enzyme activity was calculated, and the statistical test was performed via GraphPad Prism 10.2 software via one-way ANOVA.

### 4.6. Evaluation of the Effects of pH and Temperature on Enzyme Activity

To test the effect of temperature on enzyme activity, 50 µL of the PBS–caffeine enzyme mixture was incubated with 50 µL of the reaction mixture (140 mM Tris-Cl, pH 7.4, 50 mM MgCl_2_, 6.25 mM substrate, and 5 Eu/mL endotoxin) in four 96-well plates. The plates were placed into four plate readers set at 25 °C, 30 °C, 37 °C, and 42 °C. All these plate readers were validated and calibrated once a month to ensure that their test results were similar. The chromogenic assay was performed for 1 h. To evaluate the effect of pH on the activity of the PBS–caffeine enzymes, fifty microliters of the PBS–caffeine enzymes were incubated with 10 µL of buffer at six different pH values (200 mM acetate, pH 4.65; 200 mM acetate, pH 5.6; 200 mM MES, pH 6.5; 200 mM Tris-Cl, pH 7.4; 200 mM Tris-Cl, pH 8.2; and 200 mM Tris-Cl, pH 8.5) in a 96-well plate at room temperature for 5 min, after which 40 µL of each reaction mixture (50 mM MgCl_2_, 6.25 mM substrate, and 5 Eu/mL endotoxin) was added. The plate was subsequently placed in a plate reader at a reaction temperature of 37 °C. The chromogenic assay mixture was monitored at 405 nm for 1 h.

### 4.7. Evaluation of the Effects of Ions on Enzyme Activity

To test the effect of NaCl on enzyme activity, the PBS–caffeine enzyme mixture was incubated with 0, 50, 100, 150, or 200 mM NaCl in 96-well plates at room temperature for 5 min, after which 50 µL of the reaction mixture (140 mM Tris-Cl, pH 7.4, 6.25 mM substrate, and 5 Eu/mL endotoxin) was added. To test the effect of CaCl_2_ on enzyme activity, the PBS–caffeine enzyme mixture was incubated with 0, 25, 50, 100, 150, or 200 mM CaCl_2_ in 96-well plates at room temperature for 5 min, after which 50 µL of the reaction mixture (140 mM Tris-Cl, pH 7.4, 6.25 mM substrate, and 5 Eu/mL endotoxin) was added. The plate was placed in a plate reader, and the reaction was monitored at 37 °C for 1 h. To test the effect of MgCl_2_ on enzyme activity, the PBS–caffeine enzyme mixture was incubated with 0, 5, 10, 20, 30, 40, 50, 60, 70, 80, 90, and 100 mM MgCl_2_ in 96-well plates at room temperature for 5 min, after which 50 µL of the reaction mixture (140 mM Tris-Cl, pH 7.4, 6.25 mM substrate, and 5 Eu/mL endotoxin) was added. The plate was placed in a plate reader, and the reaction was monitored at 37 °C for 1 h.

### 4.8. Evaluation of the Effect of CaCl_2_ in PBS–Caffeine Buffer on Degranulation During the Blood Collection Process

To test whether CaCl_2_ could block the effect of PBS–caffeine buffer on degranulation during the blood collection process, the blood of six crabs was collected in PBS–caffeine buffer or PBS–caffeine buffer supplemented with 50 mM CaCl_2_. The blood buffer mixture was treated as described above, and the cell pellets were washed twice with 3% NaCl and finally resuspended in 5 mM CaCl_2_ resuspension solution. The cell pellet mixture was shaken at 4 °C overnight. The enzyme activity of the supernatant was tested via the chromogenic method. To test whether MgCl_2_ could block the effect of PBS–caffeine buffer on degranulation during the blood collection process, the blood of three crabs was collected in PBS–caffeine buffer or PBS–caffeine buffer supplemented with 50 mM MgCl_2_. The blood buffer mixture was treated as described above, and the cell pellets were washed twice with 3% NaCl and finally resuspended in 5 mM CaCl_2_ resuspension solution. To further test whether 100 mM CaCl_2_ could block the effect of PBS–caffeine buffer on degranulation during the blood collection process, the blood of three crabs was collected in PBS–caffeine buffer or PBS–caffeine buffer supplemented with 100 mM CaCl_2_. The blood buffer mixture was treated as described above, and the cell pellets were washed twice with 3% NaCl and finally resuspended in 5 mM CaCl_2_ resuspension solution. The cell pellet mixture was shaken at 4 °C overnight. The enzyme activity of the supernatant was tested via the chromogenic method.

### 4.9. Lyophilization Preparation of the LAL Reaction Mixture

After the LAL mixture was incubated at 4 °C for two weeks, to test whether the LAL of PBS–caffeine works in turbidimetric assays and to determine the effect of NaCl on turbidimetric parameters, 6 mL of PBS–caffeine LAL was incubated with 240 µL of 1 M MgCl_2_ for 10 min and divided into two parts, 3 mL each. One hundred microliters of 30% NaCl was added to one part of the LAL mixture, and 100 µL of LAL reagent water was added to the other. The mixture was shaken at room temperature for 10 min and then centrifuged at 3000 rpm (1650× *g*) for 10 min. Next, 2.5 mL of the supernatant was transferred to a 10 mL sterile glass vial for lyophilization at −50 °C.

### 4.10. Turbidimetric Test

The turbidimetric assay was carried out after LAL was lyophilized. The tube-based reaction mixtures comprised 100 µL of a solution containing 160 mM Tris-Cl (pH 7.4) and 0.0156, 0.031, 0.062, 0.125, 0.25, 0.5, 1, or 2 EU/mL endotoxin. One vial of lyophilized LAL was reconstituted with 2.5 mL of LAL reagent water (LRW). After gently shaking, one hundred microliters of lyophilized LAL were added to the reaction mixture. These endotoxin concentrations were halved in the final LAL-containing reaction mixture. The mixture was vortexed for 30 s before being placed in a 96-well PK Flex tube reader. The reaction was monitored at 660 nm for 120 min.

### 4.11. Statistical Analysis

*t*-tests were conducted via GraphPad Prism 10.2 software to compare the activity of the citric acid-derived LAL with that of the 3% NaCl-derived LAL, the malic acid–LRW-derived LAL with malic acid–1 mM CaCl_2_-derived LAL, the PBS-derived LAL with that of the 3% NaCl-derived LAL, the PBS–caffeine-derived LAL with that of the 3% NaCl-derived LAL, the PBS–caffeine-derived LAL with that of the PBS–caffeine–50 mM CaCl_2_-derived LAL, the PBS–caffeine-derived LAL with that of the PBS–caffeine–100 mM CaCl_2_-derived LAL, and the PBS–caffeine-derived LAL with that of the PBS–caffeine–50 mM MgCl_2_-derived LAL at a significance level of 0.05. One-way ANOVA was conducted via GraphPad Prism 10.2 software to compare the enzyme activity of the PBS–caffeine-derived LAL in LRW, 5 mM CaCl_2_, 5 mM MgCl_2_, 5 mM NaCl four different resuspension solutions at a significance level of 0.05.

## Figures and Tables

**Table 1:** Turbidimetric activity test of different LALs (PK Flex tube reader, 660 nm for 3 h). LAL was aged for 21 days at 2–8 °C.

Endotoxin (Eu/mL)	Onset Time (Sec)					
	PBS-1-LAL	PBS-3 LAL	Citric-1 LAL	Citric-2-LAL	Malic-1 LAL	Malic-2 LAL
LRW	>10,800	>10,800	>10,800	>10,800	>10,800	>10,800
0.0078	>10,800	>10,800	>10,800	>10,800	>10,800	>10,800
0.0156	5610	5122	>10,800	9693	6044	4999
0.031	2219	2185	6423	6304	2880	2780
0.062	1398	1432	2777	4355	1966	1900

The reaction mixture (0.07 M Tris, pH 7.4; endotoxin: 0.0078–0.062 EU/mL) was used. LRW—LAL reagent water.

## Data Availability

All the data generated or analyzed during this study are included in this published article.
